# OSAS-Related Inflammatory Mechanisms of Liver Injury in Nonalcoholic Fatty Liver Disease

**DOI:** 10.1155/2015/815721

**Published:** 2015-03-19

**Authors:** Elena Paschetta, Paola Belci, Anna Alisi, Daniela Liccardo, Renato Cutrera, Giovanni Musso, Valerio Nobili

**Affiliations:** ^1^Gradenigo Hospital, University of Turin, Corso Regina Margherita, 10132 Turin, Italy; ^2^Department of Medical Sciences, San Giovanni Battista Hospital, University of Turin, Corso Bramante 14, 10124 Turin, Italy; ^3^Hepato-Metabolic Disease Unit, Bambino Gesù Children's Hospital and IRCCS, S. Onofrio Square 4, 00165 Rome, Italy; ^4^Pneumology Unit, Sleep and Noninvasive Ventilation Laboratory, Bambino Gesù Children's Hospital and IRCCS, S. Onofrio Square 4, 00165 Rome, Italy

## Abstract

Obstructive sleep apnoea syndrome (OSAS) is a common sleep disorder, affecting over 4% of the general population, and is associated with metabolic syndrome and cardiovascular disease, independent of obesity and traditional risk factors. OSAS has been recently connected to nonalcoholic fatty liver disease (NAFLD), the most common chronic liver disease in the world, which can be found in 30% of the general adult population. Several studies suggest that the chronic intermittent hypoxia (CIH) of OSAS patients may per se trigger liver injury, inflammation, and fibrogenesis, promoting NAFLD development and the progression from steatosis to steatohepatitis, cirrhosis, and hepatocellular carcinoma. In NAFLD patients, liver disease may be caused by hypoxia both indirectly by promoting inflammation and insulin resistance and directly by enhancing proinflammatory cytokine production and metabolic dysregulation in liver cells. In this review, we focus on molecular mechanisms linking OSAS to NAFLD, including hypoxia inducible factor (HIF), nuclear factor kappa B (NF-*κ*B), YKL-40, unfolded protein response, and hypoxic adipose tissue inflammation, which all could provide novel potential therapeutic approaches for the management of NAFLD patients with OSAS.

## 1. Introduction

Obstructive sleep apnoea syndrome (OSAS) is a common sleep disorder in which complete or partial airway obstruction, caused by pharyngeal collapse during sleep, determines loud snoring or choking, frequent awakenings, disrupted sleep, and excessive daytime sleepiness. OSAS affects over 4% of the general population and 35–45% of obese individuals [1, 2]. The growing clinical relevance of OSAS is due to its emerging association with diabetes mellitus, metabolic syndrome, and cardiovascular disease (CVD), independent of other traditional cardiometabolic risk factors and obesity [3–6]. A lot of evidence suggests that the pathophysiological alteration in gas exchange (repetitive hypoxemic and hypercapnic events), called chronic intermittent hypoxia (CIH), can lead to increased proinflammatory cytokine production, endothelial dysfunction, oxidative stress, metabolic dysregulation, and insulin resistance [7–9]. In the latest years, OSAS has been associated with nonalcoholic fatty liver disease (NAFLD). Experimental evidence suggests that CIH may per whole trigger liver injury, inflammation, and fibrogenesis [10], and, interestingly, OSAS is also believed to be one of the elements promoting the evolution of NAFLD from steatosis to nonalcoholic steatohepatitis (NASH) [11]. NAFLD is the most common chronic liver disease in the world: about 30% of the general adult population and up to 60–70% of diabetic and obese patients suffer from this clinical condition [12]. NAFLD includes a group of liver damages, ranging from simple steatosis to NASH. NAFLD confers an increased risk of liver-related complications (hepatocellular carcinoma and end-stage liver disease), cardiovascular disease, and chronic kidney disease and is projected to be the leading cause of liver transplantation by 2020 [13, 14]. The metabolic alterations connecting to NASH encompass insulin resistance and obesity, but mechanism(s) whereby repeated hypoxic events of OSAS can enhance liver disease progression is not completely clear.

This review focuses on molecular mechanisms linking OSAS to liver injury in NAFLD.

## 2. Chronic Intermittent Hypoxia and Fatty Liver Disease

CIH is a key feature in the pathophysiology of OSAS. The mechanism is probably similar to ischemia-reperfusion injury. In OSAS patients, some oxidative stress markers are augmented and they could increase inflammation, endothelial dysfunction, and development of atherosclerosis [15].

Recently, some studies have focused on the effects of hypoxia on metabolic pathways and on mechanisms of cell injury in NAFLD. Savransky et al. showed that CIH induces hyperglycemia and hepatic lipid peroxidation and enhances activity of nuclear factor kappa B (NF-*κ*B), a master regulator of inflammatory response. Liver histology is characterized by swelling and significant increase in accumulation of glycogen in hepatocytes. Therefore, CIH may independently drive to mild liver injury also in the absence of factors that induce obesity [16]. Murine models have demonstrated that CIH leads to a significant rising in hepatic lipid peroxidation, *α*1-collagen mRNA and amount of myeloperoxidase, and proinflammatory cytokines (such as IL-1*β*, IL-6, the chemokine macrophage inflammatory protein-2, and TNF-*α*) [17]. The authors suggest that, in the chronic hypoxic conditions associated with OSAS, a high-fat diet could promote NAFLD. It has been demonstrated that hypoxia reduces insulin sensitivity in mice and could enhance expression of the lipogenic transcription factors sterol-regulatory-element binding protein-1c (SREBP-1c), peroxisome proliferator-activated receptor-*γ* (PPAR-*γ*), acetyl-CoA carboxylase 1 (ACC1), and acetyl-CoA carboxylase 2 (ACC2).

Enhanced SREBP-1c activity involves two mechanisms: activation of SREBP-1c transcription and stimulation of proteolytic cleavage of the SREBP-1c precursor form embedded in the membranes of the endoplasmic reticulum (ER) [18]. Within the ER membranes, the inactive SREBP proteins are associated with two proteins with a pivotal role in the control of the cleavage process: SREBP cleavage-activating protein (SCAP) and insulin-induced gene (*INSIG*). SCAP interacts with both newly synthesized SREBP precursor and Insig, which keep the SCAP/SREBP complex into the ER [19]. In the presence of specific signals such as insulin, SCAP dissociates from Insig and escorts SREBPs in coated protein II (COPII) vesicles from the ER to the Golgi apparatus, where SREBPs are proteolytically processed to yield the transcriptionally active form. The mature SREBP forms are released and translocate into the nucleus and the SREBP-1c mature isoforms stimulate the expression of lipogenic genes. Mounting animal and human evidence suggests that abnormalities of SREBP-1c function play an important pathogenetic role in contributing to the NAFLD phenotype [20, 21]. Insulin-resistant ob/ob mice have increased concentrations of SREBP-1c and also develop spontaneous fatty liver [22]: SREBP-1c activates, among other genes, ACC that produces malonyl-CoA, an intermediate in fatty acid synthesis, which inhibits CPT-1, the enzyme transferring fatty acyl-CoAs into the mitochondria for *β*-oxidation. PPAR-*γ* is necessary for regulation of insulin sensitivity and lipid metabolism. The overexpression of PPAR-*γ* in liver tissue causes lipid accumulation; it could represent a mechanism for hypoxia-induced fatty liver [23]. Moreover, hypoxia also reduces the expression of genes regulating mitochondrial *β*-oxidation (e.g., PPAR-*α* and carnitine palmitoyltransferase-1 (CPT-1)), which might decrease fat oxidation and promote lipid accumulation [11]. PPAR-*α* is highlyexpressed in the liver and mice lacking PPAR-*α* develop steatosis [24]. Moreover, PPAR-*α* has anti-inflammatoryproperties. PPAR-*α* suppresses the expression of proinflammatory genes, allowing the control and inhibition of inflammation [25]. Therefore, hypoxia per se can upregulate the expression of lipogenic genes and downregulate genes involved in lipid metabolism: it promotes hepatic triglyceride accumulation, necroinflammation, and fibrosis that promote the progression of NAFLD [26]. Consistent with experimental data, Nobili et al. found in paediatric NAFLD thatthe presence of OSAS was associated with the presence of NASH and of significant fibrosis, and the severity of sleep apnoea and nocturnal hypoxemia correlated with NAS score and fibrosis stage, independently of overall/abdominal obesity, metabolic syndrome, and insulin resistance [27]. In a population of obese children and adolescents with liver biopsy-proven NAFLD, Sundaram et al. have demonstrated that histological fibrosis was more severe in the subjects with NAFLD and OSA/hypoxemia compared with those without OSA/hypoxemia. Moreover, in this study, the severity and the duration of nocturnal hypoxemia were associated with both histological measures of NAFLD disease severity and elevated AST and ALT levels [28].

**Figure 1:**
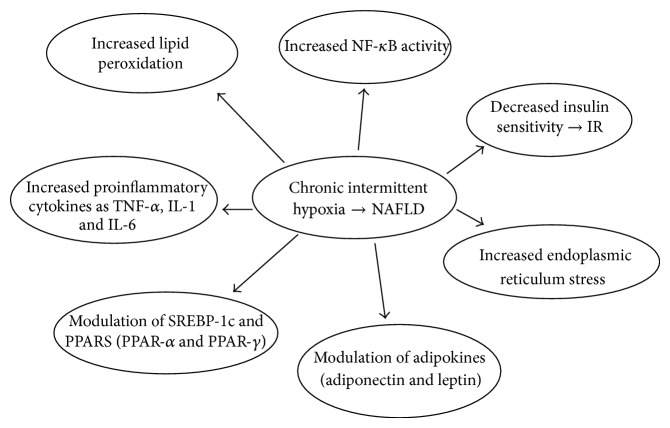
The relationship between nonalcoholic fatty liver disease (NAFLD) and chronic intermittent hypoxia (CIH). TNF: tumor necrosis factor. IL: interleukine. SREBP-1c: sterol-regulatory-element-binding protein-1c. PPAR: peroxisome proliferator-activated receptor. ER: endoplasmic reticulum.

The duration of nocturnal haemoglobin desaturation independently predicted the number of liver-infiltrating leukocytes and activated Kupffer cells/macrophages, which are believed to play a key role in the pathogenesis of liver injury in NAFLD [29]. Furthermore, CIH directly activates hypoxia-inducible factor- (HIF-) 1a and HIF-2a, two key transcription factors regulating the expression of genes involved in hepatocyte de novo lipogenesis and free fatty acid oxidation and in Kupffer and hepatic stellate cell activation, eventually promoting hepatic steatosis, necroinflammation, and fibrogenesis [30, 31] (Figure 1).

## 3. Nuclear Factor Kappa B (NF-*κ*B): A Link between OSAS and Hypoxic Liver Injury

The NF-*κ*B family of transcription factors is constitutively expressed in all cell types, which has a central role as a transcriptional regulator in response to cellular stress. The two known pathways for NF-*κ*B activation are the canonical (classical) Toll-like receptor signaling and the noncanonical (alternative) pathway which is particularly important in B cells. NF-*κ*B exists as homodimer/heterodimer composed of members of the Rel protein family which includes RelA (p65), p50 and its precursor p105 (NF-*κ*B1), and RelB, cRel, and p52 and its precursor p100 (NF-*κ*B2) in the alternative pathway. Inactive NF-*κ*B dimers are stored in the cytoplasm, under inhibition control of IkB (inhibitor of NF-*κ*B) family which bind to NF-*κ*B and masks its nuclear localization signal [32]. IkB proteosome degradation occurs by phosphorylation via the activity of IkB kinases, IKK*α*, and IKK*β*. In the classical pathway, proinflammatory cytokines like TNF*α* or oncogenes promote a kinase signalling cascade, leading to the phosphorylation of IkB and ubiquitination-mediated proteasomal degradation; finally, NF-*κ*B is released and translocates into the nucleus. In the alternative pathway, activation of IKK*α* phosphorylates NF-*κ*B precursors (p100/RelB); the proteasome then processes these precursors into the active p52/RelB heterodimer. Then, the activated NF-*κ*B dimer affects the expression of genes involved in immune responses, proliferation, apoptosis, and expression of certain viral genes by binding to target DNA sequences [33]. NF-*κ*B participates in the initiation and the progression of inflammation. Particularly, the cardiovascular and adipose tissue inflammation in OSAS seems to be related to an enhanced NF-*κ*B activity in endothelial cells, adipose tissue macrophages, and adipocytes [34]; furthermore, NF-*κ*B activation in hepatocytes and in stellate cells is associated with hepatic insulin resistance, hepatocyte apoptosis, and development of NASH and hepatocellular carcinoma in animals and humans [35, 36]. To date, systemic activation of NF-*κ*B has been related to OSAS pathophysiology independently of HIF by novel mechanism activated in hypoxic conditions [37]. In hypoxic conditions, calcium is released from the endoplasmic reticulum (ER) and activates calcium-dependent IKK kinase transforming growth factor *β* activated kinase- (TAK-) 1; TAK-1 phosphorylates I*κ*B*α*, thereby promoting RelA subunit release and NF-*κ*B activation and translocation into the nucleus. Hypoxia also inhibits specific sumo proteases (Senps), resulting in an increased Sumo-2/3 (S2) chains on IkB*α*, which is sufficient to release RelA from IkB*α* and activate NF-*κ*B [38]. In addition, NF-*κ*B pathway is linked to oxidative stress and reactive oxygen species (ROS) by the production of inducible NO synthase (iNOS), cyclooxygenase- (COX-) 2, and metalloproteinase 9. ROS released from hypoxic hepatocyte have been shown to directly activate hepatic stellate cell through IkB-*α* phosphorylation and NF-*κ*B signalling activation in cell culture [39]. The induction of NF-*κ*B by CIH may lead to upregulation of multiple inflammatory cytokines and chemokines, including IL-1*α*, IL-1*β*, IL-2, IL-4, IL-6, IL-8, I-10, IL-13, IL-15, IL-18, TNF-*α*, TNF-*β*, IFN-*α*, IFN-*β*, and macrophage inhibitory protein 1b [40].

The unfolded protein response (UPR) represents another mechanism linking hypoxia and liver injury. A pathogenic role of UPR in steatosis, inflammation and insulin resistance is supported by studies on obese and nutritional models of fatty liver [41] and on NAFLD patients [42].

The ER is a specialized cellular organelle synthesizing, folding, and assembling membrane and secretory proteins. Any condition perturbing ER homeostasis, as excessive protein synthesis or alterations in cellular redox balance or in calcium concentration, triggers a physiologic response, the UPR, which involves an increment of ER-folding capacities by increasing the transcription of ER-resident chaperones and protein foldases, to a downregulation of the protein load in the ER lumen by reducing protein synthesis and to an ER-associated degradation (ERAD) of irretrievably misfolded proteins [43].

The UPR is mediated by 3 transducer proteins that are integral membrane proteins of the ER: inositol-requiring kinase-1 (IRE1), activating transcription factor 6 (ATF6), and protein kinase RNA-like ER kinase (PERK), which are normally maintained inactive by the linking of intraluminal ER chaperones, including the binding immunoglobulin protein (BiP)/glucose-regulated protein 78 (GRP78). During UPR, BiP is sequestered by the unfolded proteins and it dissociates from the three ER-transmembrane transducers, leading to their activation. IRE1*α* is an inositol-requiring enzyme that regulates the expression of the transcription factor X box-binding protein 1 (XBP1) and regulates the activity of kinase c-Jun N-terminal kinase (JNK). ATF6 is a transcription factor that, like SREBP-1c and SREBP-2, translocates to the nucleus, upregulating chaperones/foldases such as GRP78, homocysteine-induced ER protein (HERP), calreticulin, and calnexin, which enhance the folding ability of the ER. PERK phosphorylates eukaryotic initiation factor 2*α* (eIF2*α*) causing a global mRNA translation attenuation and, at the same time, selectively enhances the translation of several mRNAs, including the activating transcription factor 4 (ATF4), which upregulates chaperones and antioxidant response genes and increases damaged ER repairing [44].

When the mechanisms of adaptation are saturated, the ER-folding capacity cannot be restored and the UPR over-activation results in pathologic conditions, such as the trigger of apoptosis through the activation of transcription factor C/EBP homologous protein (CHOP), of JNK, and of caspases [45].

Recent evidence has shown that UPR is a component of the cellular response to hypoxia and PERK has been identified as the kinase responsible for elf-2*α* phosphorylation in hypoxic conditions [46].

Several recent studies have linked the UPR to lipogenesis regulation and hepatic steatosis.

The degree of UPR contribution to hepatic steatosis may depend on the relative activation of the 3 transducer proteins IRE1a, PERK, and ATF6. IRE1a-dependent activation of JNK can lead to liver damage and hepatocyte apoptosis, a characteristic feature of NAFLD [47].

PERK-dependent factor Nrf2 transcription is part of an antioxidant pathway. In murine model, Nrf2 deletion results in rapid onset and progression of NASH. These data suggest that PERK plays a critical role in the defence against oxidative stress linked to NASH [48].

Different studies have shown that ATF6 can inhibit the transcriptional activity of SREBP2, regulating lipid storage in the liver [49].

To date, different experimental mice models have demonstrated that CIH leads to increased phosphorylation of PERK, pointing to an upregulation of the UPR in liver and adipose tissue, collectively suggesting that ER stress may be a key mediator of hypoxia-induced liver injury and NAFLD.

Extensive evidence supports the notion that metabolically active adipose tissue plays a role in the development of NASH through altered secretion of lipotoxic free fatty acids and of adipocytokines including adiponectin, leptin, TNF*α*, or IL-6 [50].

In obese patients, adipocytes dysfunction, apoptosis, and consequent macrophage accumulation are associated with local hypoxemia [51] supporting the idea that adipose tissue hypoxia is one of the causes of adipose tissue inflammation. Evidences demonstrated that, in hypoxic condition, adipocytes express more proinflammatory cytokines (i.e., leptin, TNF*α*, and IL-6) and less adiponectin than in normoxia [52]. In adipocytes, hypoxia reduces adiponectin secretion, directly or indirectly through TNF*α* stimulation [53], and stimulates lipolysis and inhibits uptake of FFA through a direct inhibitory effect on the fatty acid transporters (FATP1 and CD36) and on the transcription factor (PPAR-*γ*) [54]. From these data, one might assume that OSAS can associate with obesity making the adipocyte prone to dysfunction and death, aggravating liver and metabolic disease.

The concentration of TNF-*α* correlates with the severity of OSAS. TNF-*α* has a role in modulation of physiological sleep. Independently of obesity, in OSAS subjects, TNF-*α* is higher than in healthy population and its levels are lower after the introduction of CPAP therapy [55]. Furthermore, in OSAS patients, the secretion rhythm of TNF-*α* is changed: the nocturnal peak of secretion is substituted by an abnormal daytime peak [56].

During nocturnal hypoxia, adipocytes and circulating monocytes secrete IL-6 through the NF-*κ*B pathway. IL-6 represents an important stimulus of CRP production in the liver [57] and has a role in inflammatory processes in sleep disorders. An increase of IL-6 has been shown in OSAS patients as compared to healthy controls [58], but IL-6 circadian rhythm is not altered in OSAS differently from TNF-*α* [59].

The increase in TNF-*α* and IL-1 is shown not only in OSAS. It is associated with an increase in insulin resistance [68], metabolic syndrome, and obesity [37]. The relationship between OSAS and CRP in obesity remains somewhat controversial, mainly, because of the major confounding effect of BMI. Nevertheless, several authors demonstrated that, in OSAS patients, CRP levels are independently associated with the severity of the sleep disturbance and of nocturnal hypoxemia independently of adiposity [69–71].

Adiponectin is another cytokine produced by adipocytes. The presence and severity of NAFLD are correlated to decreased adiponectin [72] and recently several authors have demonstrated that, in adipose tissue, the expression of adiponectin is decreased by hypoxia [73]; the consequence could be the increased expression of inflammatory cytokines. Moreover, TNF-*α* has been shown to inhibit adiponectin in adipose tissue [74]. Collectively, these data suggest that hypoxia might directly or indirectly inhibit adiponectin expression.

Leptin is a cytokine produced by adipocytes that have a role in the regulation of food intake, lipid and glucose metabolism, and the energy balance. In a rabbit model of OSAS, leptin activated inflammation and mediates cellular injury in association with IH [75]. It is known that the increased leptin production is associated with visceral obesity; also hypoxia can stimulate leptin production [76]. In several studies leptin is linked with OSAS independently of obesity [77]. Kapsimalis et al. confirmed that nocturnal hypoxemia is associated with leptin independently of obesity [71].

## 4. Hypoxia-Inducible Factors: A Key Factor of Hypoxia-Mediated Steatosis and Inflammation in the Liver

Hypoxia modulates target genes expression through a number of transcription factors, including hypoxia-inducible factors (HIFs). HIFs are heterodimers consisting of an *α* and a *β* subunit. There are three *α* subunits: the hypoxia-inducible factor 1*α* (HIF1*α*), the hypoxia-inducible factor 2*α* (HIF2*α*), and the hypoxia-inducible factor 3*α* (HIF3*α*); HIF*α* subunits bind to a common *β* subunit called or aryl- hydrocarbon receptor nuclear translocator (ARNT) [78].

HIF-*α* subunits are constitutively produced; in normoxic cells, HIF-*α* subunits are immediately degraded through hydroxylation by three prolyl-hydroxylases (PHD1, PHD2, or PHD3). The proline residues hydroxylated are assembled on a multimeric protein complex that includes the Von Hippel-Lindau (VHL) protein. This leads to rapid ubiquitination and proteasomal degradation of HIF-*α* [79]. Under hypoxic conditions, the mechanism of degradation is inhibited and HIF-*α* subunits heterodimerize with HIF-1*β*; active HIF translocates to the nucleus and binds to the hypoxia responsive elements (HRE) of hundreds of hypoxia responsive genes, regulating gene transcription [80]. In addition, HIFs can modulate target gene expression causing chromatin conformational changes [81].

In OSAS patients, the chronic intermittent cycles of hypoxia and reoxygenation (CHI) can increase active HIFs levels. In animal model, CHI leads to hypercholesterolemia and hepatic lipid peroxidation, in the absence of obesity [61].

HIFs could play a role in the pathogenesis of NAFLD, affecting many metabolic pathways in hepatic cells (see Table 1). The increase of HIF1 and HIF2 is associated with the expression in the hepatocytes of genes important for lipogenesis and gluconeogenesis regulation, triglycerides storage, and fatty acid synthesis, uptake, and *β*-oxidation. HIF1 is a mediator of alcohol-induced lipid accumulation as part of the monocyte-chemoattractant protein-1 (MCP-1) pathway; a constitutive activation of HIF2 leads to an increase of hepatic fatty acid uptake and lipid storage, along with a reduction in fatty acid *β*-oxidation and in lipoprotein lipase activity [30, 60, 62].

In hepatocytes, HIFs also regulate the transcription of genes involved in inflammation and HIF2 overexpression stimulates proinflammatory cytokines synthesis (IL-1*β* and IL-6) in hepatocytes and in macrophages [30].

Interestingly, HIFs can modulate fibrogenesis and angiogenesis in hepatic Kupffer and stellate cells [30, 31, 65]. Hypoxic HIF1 level in stellate cells is both associated with upregulation of proangiogenic mediator, including vascular endothelial growth factor (VEGF) and placental growth factor (PGF), and macrophage migration inhibitory factor (MIF) release, which is implied in fibrosis development. In Kupffer cells, HIF1 activation leads to production of platelet-derived growth factor- (PDGF-) B, VEGF, and Angiopoietin-1, promoting fibrosis and neoangiogenesis. Furthermore, HIF-2 coordinates the reprogramming of cell metabolic pathways from aerobic to anaerobic and generates ATP in an oxygen-independent manner [82]. These observations could link the increases of HIF levels not only to NAFLD development and progression but also to the pathogenesis of hepatocellular carcinoma (HCC).

In animal models, the overexpression of HIF1*α* and HIF2*α* has been found to be related to hepatic steatosis, hepatitis, and fibrosis. Hepatocyte-specific deletion of HIFs protected from steatohepatitis, whereas disruption of VHL resulted in a robust accumulation of lipids in the liver and an increase in liver inflammation and fibrosis [30, 31, 60–62].

Collectively, these data suggest that activation of HIFs in chronic hypoxemic conditions could cause hepatic steatosis, inflammation, and fibrosis, leading to NAFLD and HCC development; HIFs pathway may represent a novel potential therapeutic target.

## 5. YKL-40: A Novel Biomarker of Inflammation

YKL-40, a member of the mammalian chitinase-like proteins, is a glycoprotein consisting of 383 amino acids with a molecular mass of 40 kDa [83]. YKL-40 is secreted by activated macrophages, neutrophils, and vascular smooth muscle cells during inflammation. YKL-40 represents a biomarker of systemic inflammation and high YKL-40 levels were found in numerous pathological conditions, such as atherosclerosis, diabetes, obstructive lung disease, asthma, liver fibrosis, inflammatory bowel disease, rheumatoid arthritis, and cancer [84].

In human liver, YKL-40 may be involved in extracellular matrix turnover and its expression is regulated by TNF*α*, through the modulation of upstream transcriptional complexes that interact with the YKL-40 promoter. Sarma et al. have identified the putative binding sites for NF-*κ*B subunit P65 and CCAAT/enhancer-binding protein alpha (CEBP*α*) in the YKL40 promoter, demonstrating that the TNF mediated YKL-40 expression involves the NOTCH/NFKB signaling pathway [85].

YKL-40 has been found to be upregulated in alcoholic hepatitis and HCV patients [86, 87], and serum YKL-40 has been shown to be associated with degree of fibrosis progression and extracellular matrix synthesis in many chronic liver diseases [88]. However, in 95 patients with nonalcoholic fatty liver disease, Malik et al. observed that YKL-40 performed relatively poorly as marker of liver inflammation or fibrosis [89]; moreover, in 52 children with biopsy-verified NAFLD, Lebensztejn and coworkers did not find a correlation between YKL-40 and fibrosis stage [90].

In OSAS patients, Xang et al. observed a significant association between increments in serum YKL-40 concentrations and severity of OSAS; the YKL-40 levels were found independently correlated with AHI scores. In these patients, YKL-40 is probably secreted from activated inflammatory cells located in the upper airway or synthesized and released from differentiated VSMCs in response to tissue remodelling. Macrophages exposed to YKL-40 increase the release of proinflammatory and profibrogenic mediators, such as IL-8, IL-18, macrophage inflammatory protein- (MIP-) 1, matrix metallopeptidase- (MMP-) 9, and monocyte chemotactic protein- (MCP-) 1. This mechanism may contribute to OSAS development and progression [84].

In conclusion, YKL-40 expression is enhanced in numerous local inflammatory processes, including OSAS and chronic liver diseases, but the role of this protein in NAFLD development and progression needs to be clarified; moreover, further studies are needed to elucidate if the OSAS-dependent YKL-40 increase could trigger liver injury or affect the progression of a concomitant liver disease.

## 6. Insulin Resistance (IR) and Liver Injury in OSAS 

Hypoxia can cause insulin resistance and inflammation causing development of NAFLD. In humans, OSAS has been known as a risk factor for insulin resistance, independently of obesity [91]. In OSAS, glucose intolerance correlates positively with severity of the disease. Different studies have shown that CIH and sympathetic nerve discharge are a possible cause of alteration in insulin sensitivity. In healthy men undergoing an euglycaemic hyperinsulinemic clamp test, a short (30-minute duration) period of hypoxia was able to induce glucose intolerance and to increase plasma epinephrine levels [92].

Hypoxia can inhibit insulin receptor activation and trigger the formation of inflammatory cytokines which promote peripheral insulin resistance, while sympathetic activation causes insulin resistance by inducing glycogenolysis and gluconeogenesis [93].

It is certain that hypoxia inhibits respiratory function and biogenesis of the mitochondria and has also been found to decrease the number of mitochondria, alterations that have been linked to both insulin resistance and NAFLD [94, 95]. Hypoxia is identified to induce ER stress and inhibition of ER stress protects mice against insulin resistance and NAFLD [96].

In vitro exposure of human and murine adipocytes to prolonged hypoxia decreased phosphorylation of IRS-1 and IRS-2 and induced IR [97]. Furthermore, exposure to continuous hypoxia causes multiple changes in cell metabolism, including a switch to anaerobic glycolysis. In adipocytes, continuous hypoxia increased the expression of the glucose transporter- (GLUT-) 1 [98], glucose uptake, and release of lactate but decreased the expression of the insulin-dependent glucose transporter- (GLUT-) 4 [99].

The loss of the repressive effect of insulin on the expression of CYP2E1 could be a possible link between OSAS, insulin resistance, and NASH [100, 101]. CYP2E1 is a major microsomal source of oxidative stress and it could play a role in the pathogenesis of NASH [102]. Increased hepatocyte CYP2E1 expression may also result in the downregulation of insulin signalling, potentially contributing to the insulin resistance associated with NAFLD [103]. In the liver of mice exposed to hypoxia CYP2E1, mRNA and protein levels were increased. However, in the future, other studies are needed to elucidate if increased CYP2E1 is a cause or a consequence of insulin resistance.

## 7. Conclusion

Several studies correlate OSAS with liver inflammation and they suggest that CIH may play a role in the pathogenesis of NAFLD and in the progression from steatosis to steatohepatitis, cirrhosis, and liver cancer.

OSAS and obesity often coexist and share common molecular mechanisms that lead to metabolic disease, which could represent potentially therapeutic targets; in the future, it will be important to clarify the relative importance of these factors in the pathogenesis of liver disease.

## Figures and Tables

**Table 1:** Molecular targets and biological effects of hypoxia inducible factors (HIFs) in the liver.

Cell	Molecular target	Biological effect
HIF1*α*		
Hepatocyte	↑ Lipid droplet binding protein adipose differentiation-related protein (ADFP)	↑ Triglyceride storage in lipid droplets [60]
	↑ Stearoyl-coenzyme A desaturase 1 (SCD-1)	↑ De novo lipogenesis [61]
	↑ Enolase 1 ↑ Lactate dehydrogenase 1	↑ Glycolysis ↑ Pyruvate metabolism [62]
	↑ Membrane glucose transporter- (GLUT-) 1 and GLUT-3	↑ Glucose uptake [63]
	↑ Plasminogen activator inhibitor- (PAI-) 1 ↑ Platelet-derived growth factor- (PDGF-) A and PDGF-B	↑ Hepatic stellate cell activation and fibrogenesis [64]
Hepatic stellate cells	↑ PDGF-B ↑ Transforming growth factor- (TGF-) *β*1 ↑ Vascular endothelial growth factor (VEGF) ↑ Chemokine receptor- (CCR-) 1 and 5	↑ Fibrogenesis ↑ Angiogenesis ↑ Carcinogenesis [31]
Kupffer cells	↑ Plasminogen activator inhibitor- (PAI-) 1 ↑ Platelet-derived growth factor- (PDGF-) A and PDGF-B	↑ Hepatic stellate cell activation and fibrogenesis [65]

HIF2*α*		
Hepatocyte	↑ Lipid droplet binding protein adipose differentiation-related protein (ADFP)	↑ Triglyceride storage in lipid droplets [62]
	↓ Acyl-coenzyme A synthase long-chain family member 1 ↓ Carnitine-palmitoyltransferase I	↓ Mitochondrial fatty acid *β*-oxidation [62]
	↓ Acyl-CoA oxidase (Aco) ↓ Carnitine O-octanyltransferase	↓ Peroxisomal fatty acid *β*-oxidation [62]
	↑ Membrane fatty acid transporter cluster differentiation 36 (CD36)	↑ Fatty acid uptake from plasma [30]
	Sterol-regulatory-element binding protein-1c (Srebp-1c) Fatty acid synthase (Fas) Acetyl-CoA carboxylase (Acc) (↑ early after HIF2*α* activation and then ↓ after long-term HIF2*α* activation)	De novo lipogenesis (↑ early after HIF2*α* activation and then ↓ after long-term HIF2*α* activation) [30, 62]
	↑ Angiopoietin-like 3 (Angptl3)	↓ Tissue lipoprotein lipase activity ↓ ↑ Plasma triglycerides [62, 66]
	↓ Phosphoenolpyruvate-carboxykinase (Pepck) ↓ Glucose-6-phosphatase	↓ Gluconeogenesis [62, 67]
	↓ Peroxisome proliferator-activated receptor *γ* coactivator 1*α* (Pgc-1*α*) ↓ Hepatocyte nuclear factor 4 (Hnf4)	↓ Gluconeogenesis [62, 67] ↓ Mitochondrial fatty acid *β*-oxidation [62]
	↑ Interleukin-1*β* ↑ Interleukin-6	↑ Inflammation [30]
Macrophage	↑ Interleukin-1*β* ↑ Interleukin-6	↑ Inflammation [30]
Hepatic stellate cells	↑ Lysyl oxidase-like- (LOXL-) 1 and LOXL-2 ↑ Prolyl 4-hydroxylase *α*- (P4HA-) 1 and P4HA-2 ↑ Procollagen lysine ↑ 2-Oxoglutarate 5-dioxygenase 2 (PLOD2) ↑ Transglutaminase 2 (TGM2)	↑ Collagen synthesis and deposition ↑ Fibrogenesis [30]
